# Strenuous physical activity is associated with a younger age of amyotrophic lateral sclerosis onset in two independent cohorts

**DOI:** 10.1093/braincomms/fcag272

**Published:** 2026-07-13

**Authors:** Jaimee Kennedy, Ahmad Al Khleifat, Andrew King, Safa Al-Sarraj, Ammar Al-Chalabi, Jacqueline C Mitchell, Claire Troakes

**Affiliations:** Maurice Wohl Clinical Neuroscience Institute, Department of Basic and Clinical Neuroscience, King's College London, London SE5 9RT, UK; London Neurodegenerative Diseases Brain Bank, Institute of Psychiatry, Psychology & Neuroscience, Kings College London, London SE8 8AF, UK; Maurice Wohl Clinical Neuroscience Institute, Department of Basic and Clinical Neuroscience, King's College London, London SE5 9RT, UK; London Neurodegenerative Diseases Brain Bank, Institute of Psychiatry, Psychology & Neuroscience, Kings College London, London SE8 8AF, UK; Clinical Neuropathology Department, Kings College Hospital NHS Trust, London SE5 9RS, UK; London Neurodegenerative Diseases Brain Bank, Institute of Psychiatry, Psychology & Neuroscience, Kings College London, London SE8 8AF, UK; Clinical Neuropathology Department, Kings College Hospital NHS Trust, London SE5 9RS, UK; Maurice Wohl Clinical Neuroscience Institute, Department of Basic and Clinical Neuroscience, King's College London, London SE5 9RT, UK; Department of Clinical Neurosciences, King’s College Hospital NHS Trust, London SE5 9RS, UK; Maurice Wohl Clinical Neuroscience Institute, Department of Basic and Clinical Neuroscience, King's College London, London SE5 9RT, UK; Maurice Wohl Clinical Neuroscience Institute, Department of Basic and Clinical Neuroscience, King's College London, London SE5 9RT, UK; London Neurodegenerative Diseases Brain Bank, Institute of Psychiatry, Psychology & Neuroscience, Kings College London, London SE8 8AF, UK

**Keywords:** motor neuron disease, amyotrophic lateral sclerosis, physical activity, age onset, exercise

## Abstract

Amyotrophic lateral sclerosis (ALS) is a complex neurodegenerative disease characterized predominantly by degeneration of both upper and lower motor neurons, thought to occur due to an interplay between genetics and environmental factors. Physical activity has been suggested as a potential risk factor for ALS; however, the exact role of exercise in the onset and progression of the disease is still unclear.

We assessed lifetime physical activity in two independent ALS cohorts: post-mortem brain donors from the London Neurodegenerative Diseases Brain Bank (*n* = 139) and patients from the Motor Neurone Disease (MND) Register of England, Wales and Northern Ireland (*n* = 166 cases, 196 controls).

In both cohorts, highly active individuals developed ALS symptoms at a significantly younger age, 54.2 years (mean, standard deviation = 7.5) in the post-mortem cohort and 58.0 years (median, interquartile range = 15) in the MND Register, compared with 63.9 years (mean, standard deviation = 11.5) and 63.0 years (median, interquartile range = 17.5) in inactive individuals, respectively [one-way analysis of variance (ANOVA), *F*(2, 136) = 6.10, *P* = 0.003, $\eta^{2}$ = 0.08, 95% confidence interval (CI) 0.02–1.00 and Kruskal–Wallis, H(2) = 7.39, *P* = 0.02, $\eta^{2}$ = 0.03, 95% CI 0.003–0.12]. Cox regression showed a higher hazard of earlier onset in highly active patients [post-mortem: hazard ratio (HR) 2.84, 95% CI 1.55–5.26, *P* = 0.0008; MND Register: HR 2.34, 95% CI 1.30–4.23, *P* = 0.005].

Our findings suggest that strenuous physical activity may be associated with a significantly younger age of ALS onset, replicated in both the post-mortem and MND Register cohorts, but not with an increased risk of developing ALS. Logistic regression analysis confirmed that neither highly active [odds ratio (OR) 1.43, 95% CI 0.69–2.99, *P* = 0.333] nor being active (OR 1.30, 95% CI 0.72–2.37, *P* = 0.386) was significantly associated with ALS risk, whereas a history of head injury was (OR 1.72, 95% CI 1.03–2.88, *P* = 0.038). These results suggest that strenuous exercise may accelerate disease onset in predisposed individuals, while the role of head injury requires further study and the findings may in fact indicate reverse causality.

## Teaser

Kennedy *et al*. report that strenuous exercise may contribute to an earlier onset of amyotrophic lateral sclerosis in susceptible individuals. Within two cohorts, they show a significantly younger age of symptom onset in highly active individuals with amyotrophic lateral sclerosis compared to those who did not engage in noteworthy physical activity.

## Graphical Abstract

**Graphical Abstract:**
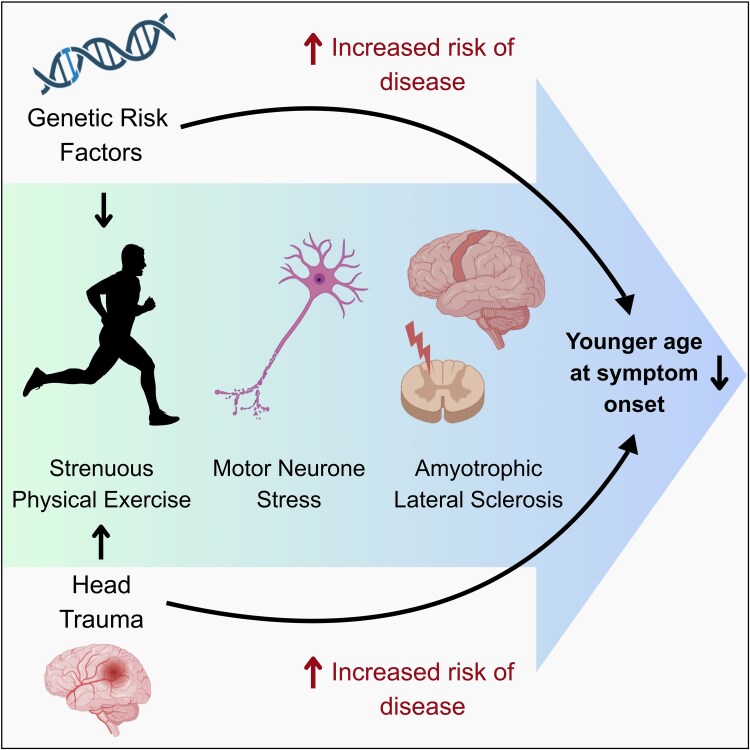

## Introduction

Amyotrophic lateral sclerosis (ALS) is a motor neuron disease (MND) characterized by progressive degeneration, predominantly of the upper and lower motor neurons, leading to progressive weakness and eventual death ∼3–5 years from disease onset.^1^ The incidence rate of ALS in Europe ranges from two to three cases per 100 000 person-years, with a lifetime risk of one in 300^2^ and recent studies estimate the mean age of disease onset is between 51 and 66 years.^3,4^ ALS is ∼1.2–1.5 times more common in men than women.^5^

Five to 10% of people with ALS have a family history, and known Mendelian variants explain ∼20% of all cases of ALS.^6-8^ The majority are apparently sporadic, with poorly understood pathophysiological mechanisms; given that heritability is estimated at ∼60%,^9^ much of the genetic contribution remains unexplained, suggesting disease likely arises from a complex interplay between unidentified genetic factors and environmental influences.^10^ Indeed, the penetrance of the most common ALS-linked mutation, intronic hexanucleotide repeat (GGGGCC) expansion of *C9orf72*, is probably around 45%, suggesting that even in the presence of an ALS risk gene, interacting environmental factors also play an important role in disease onset.^11^

Over the last 20 years, epidemiological studies have suggested several potential environmental risk factors for ALS, including smoking,^12^ head trauma,^13-16^ heavy metal exposure^17^ and exercise,^18-26^ although none has been definitively established. The confirmation of such risk factors has proven difficult, and studies are limited by a small sample size and challenges with retrospective recall of premorbid exposures.

Partaking in high levels of physical activity is a risk factor which has seen heightened interest in recent years with research suggesting an increased incidence of ALS in athletes, and not just players of contact sports, where repetitive mild head injury may play a role, but also in those undertaking regular intensive exercise. Several studies report a higher incidence and lower age of disease onset in both football and rugby players,^19-22^ as well as in sports where head injury is uncommon such as cross-country skiing.^23-25^ Moreover, a recent study has shown a decrease in age of ALS onset in carriers of the *C9orf72* expansion mutation who participate in frequent strenuous exercise, inversely proportional to levels of historical physical activity.^26^ However, the role of physical activity and its relationship to ALS is still heavily debated in the field and more research is needed to understand the complex and nuanced interaction between exercise, genetics and the onset of disease.^27^

Although it could be argued that the obvious cardiovascular health benefits of exercise outweigh the potential increased risk of a relatively rare disease such as ALS, the exploration of such lifestyle factors has implications for understanding the development of ALS and may contribute to personalized therapeutic approaches. This study aims to assess the hypothesis that intensive physical activity reduces the age of symptom onset and survival time in people with apparently sporadic ALS, within two separate cohorts: Brain Bank post-mortem ALS tissue donors and case-control participants from the MND Register of England, Wales and Northern Ireland (https://mndregister.ac.uk).^28^

## Materials and methods

### Study cohort

To investigate how physical activity may have an impact on age at symptom onset and survival probability, historical exercise data over the course of a lifetime was extracted from two ALS patient cohorts: post-mortem brain donors from the London Neurodegenerative Diseases Brain Bank and participants in the MND Register of England, Wales and Northern Ireland.^28^

The Brain Bank database was searched to reveal all tissue donors with a primary neuropathological diagnosis of ALS (with or without concurrent frontotemporal dementia) between 2001–22 (*n* = 207). All those with apparently sporadic ALS with neuropathological evidence of TDP-43 proteinopathy (including people carrying the *C9orf72* expansion) were eligible for inclusion; however, any donors with a confirmed genetic mutation in *FUS* or *SOD1* (*n* = 13), a known family history of ALS or frontotemporal dementia in a first-degree relative or primary pathology (or extensive secondary pathology) other than ALS or frontotemporal dementia such as Alzheimer’s disease (*n* = 17), were excluded. Informed consent for brain donation and access to medical records was taken under the Brain Bank’s ethical approval; 23/WA/1024, REC 3 for Wales, 17/05/2023. Medical records (including details from referral letters and from the Electronic Patient Record) were obtained from the general practitioner or consultant neurologist of each donor at the time of brain donation. Any donors with absent, very little or conflicting records were excluded (*n* = 34), in addition to donors with clinical evidence indicating primary lateral sclerosis, progressive bulbar palsy or progressive muscular atrophy rather than classical ALS (*n* = 4). Detailed information on historical leisure-time physical activity was mined from the records of the remaining cases with available medical records (*n* = 139).

The MND Register for England, Wales and Northern Ireland is a population-based record of people with MND under ethical approval from the London–South East Research Ethics Committee (REC reference 15/LO/0810) in which a subset has epidemiological information available. The data were obtained from the MND Association of England, Wales and Northern Ireland (MNDA) Collections as part of the MNDA Epidemiology Study, REC reference 07/MRE01/57. Three tertiary centres in London, Sheffield and Birmingham acted as data collection hubs but people with ALS were also recruited at secondary centres such as district general hospitals. General practitioners from the general practice of the person with ALS were asked to invite 10 healthy controls to participate in the study. The research team matched people on age (within 5 years of the person with ALS) and sex in a 1:1 ratio. Three hundred and ninety-five people participated in a telephone interview about their lifestyle including physical activity, undertaken by a trained nurse. Two participants did not provide information on physical activity, and one participant did not provide symptom onset data. Thirty further cases in the MND Collections epidemiology dataset were excluded by the same criteria outlined above for the post-mortem cohort, confirmed genetic mutation in *FUS* or *SOD1* (*n* = 1), known family history of ALS or frontotemporal dementia (*n* = 20), or a diagnosis of primary lateral sclerosis, progressive bulbar palsy or progressive muscular atrophy (*n* = 9). In total, full data was attained from 166 individuals with definite, probable or possible ALS according to the El Escorial criteria and 196 healthy controls between 2009–15, with follow-up lasting up to 10 years.

Based on the information in the medical records and questionnaire data, there are no professional athletes within either of our study cohorts, rather these are individuals undertaking recreational exercise.

### Classification and scoring of physical activities

Physical activity can be defined as any bodily movement produced by skeletal muscles that requires energy expenditure.^29^ This can be further categorized into sporting, household and occupational activities, and in the context of this study, leisure-time physical activity refers to planned and structured exercise proactively undertaken as a recreational pastime. Occupational and household activities were excluded from scoring. As an objective measure of energy expenditure, each active individual was attributed a lifetime physical activity (LPA) score using the Compendium of Physical Activities, a widely validated tool in health research, provides a comprehensive list of metabolic equivalent task (MET; standardized for resting metabolic rate as 1 MET = 3.5 mL/kg/min) values attributed to specific activities.^30,31^ Each reported leisure-time physical activity within the available medical records or questionnaire data was matched to the most appropriate corresponding activity code and subsequent MET value.

Due to variability in reporting activity frequency within medical records, only activity type and related energy expenditure was incorporated into scoring. The LPA score for an individual was calculated by totalling the MET scores of each stand-alone activity reported within the medical records over the course of a lifetime until disease onset or exercise cessation. Unsurprisingly, large variation in activity intensity was observed within the original ‘Active’ group. Subsequently, after calculation of the LPA scores, all active donors were ranked from highest to lowest with the top scoring quartile classified as ‘Highly Active’, the rest of the group labelled ‘Active’ and all ALS donors who reported no physical activity termed ‘Inactive’. Group categorization was applied separately within each cohort based on the individual scoring distribution of each cohort (post-mortem: Highly Active: >14.7, Active: 1–14.7, Inactive = 0. MND Register: Highly Active: >27.4, Active: 1–27.4, Inactive = 0).

Other demographic and lifestyle information was also extracted for both cohorts. History of head injury encompassed both repetitive and severe head injuries reported in either medical records or questionnaire data. Any evidence of more than one clinically diagnosed concussion, non-concussive impacts or participation in contact sports commonly associated with repetitive head trauma,^32,33^ fulfilled the criteria for a history of repetitive head injury. A severe head injury was defined as a single, significant head trauma with loss of consciousness.

### Statistical analysis

All statistical analysis was conducted using R Studio (Version 2024.04.2+764). Shapiro–Wilk test was used to assess whether group data was normally distributed, and homogeneity of variance was checked with Levene’s test. Categorical demographic variables were compared by $\chi^{2}chi-squared$ or Fisher’s exact test. To assess the difference in mean and median age at disease onset (first symptom onset) and disease duration (symptom onset to death) between groups, a one-way analysis of variance (ANOVA) with Tukey’s honestly significant difference *post hoc* test or Kruskal–Wallis analysis with Dunn’s *post hoc* test and a Bonferroni correction was conducted.

Effect sizes were calculated with either Cramer’s *ν* ($\chi^{2}$, Fisher’s exact) or eta-squared ($\eta^{2}$) (one-way ANOVA, Kruskal–Wallis) tests.

### Cox proportional hazards regression

Cox proportional hazards regression analysis was used to assess the effect of multiple covariates on ALS symptom onset in both cohorts. Time to event variable was defined as the time in years from birth until first ALS symptom, with the age at symptom onset being the event of interest. The model included the following covariates: physical activity group [categorical: inactive (reference), active, highly active], sex [categorical: female (reference), male], *C9orf72* repeat expansion [categorical: absent (reference), present], history of head injury [categorical: no (reference), yes] and a binary indicator for disease status [yes (1), no (0)].

A multivariable Cox proportional hazards regression was also used to examine the association between patient characteristics and overall survival for both cohorts. The time to event variable was the time from first ALS symptom to death (or last follow-up), with death being the event of interest. The model included the following covariates: physical activity group [categorical: inactive (reference), active, highly active], sex [categorical: female (reference), male], site of onset [categorical: limbic (reference), bulbar], *C9orf72* repeat expansion [categorical: absent (reference), present], history of head injury [categorical: no (reference), yes], age at symptom onset (continuous, years) and a binary indicator for survival status [yes (1), no (0)]. A directed acyclic graph (DAG) was produced to clarify the rationale behind covariate inclusion for each model (Supplementary Figs. 1–2).

An alternative Cox model including the LPA score (continuous, cumulative MET values) instead of physical activity categorical groups, was conducted for symptom onset and survival in each cohort. Multiple linear regression analysis was used to confirm any relationship between physical activity and age at symptom onset in each cohort.

Schoenfeld residuals test confirmed that all covariates met the proportional hazards assumption (*P* > 0.05). The goodness of model fit was assessed using the likelihood ratio test (*P* < 0.05) and Wald test (*P* < 0.05). Multicollinearity among the independent variables was assessed within each cohort (and for each model) using the variance inflation factor. All variance inflation factor values were below two, indicating that multicollinearity was not a concern in any model.

Thirty-seven data points in the MND Register cohort were censored (unknown if a participant had died since last follow-up).

### Logistic regression

Binomial logistic regression was conducted to assess whether highly active individuals from the case and control groups of the MND Register cohort had an increased risk of ALS. The model included the following covariates: physical activity group [categorical: inactive (reference), active, highly active], sex [categorical: female (reference), male], age at survey (continuous, years), history of head injury [categorical: no (reference)] and an outcome variable for ALS disease status [yes (1), no (0)].

All assumptions for binomial logistic regression were met. Continuous predictors had a linear relationship with the log-odds as confirmed by a Box–Tidwell test (*P* > 0.05). A variance inflation factor test confirmed that multicollinearity was not a concern among the independent variables in the case-control model (variance inflation factor values below two). R function detect_separation() confirmed no complete or quasi-complete separation within the model. A Hosmer–Lemeshow test confirmed goodness of fit (*P* > 0.05). Thirty-three individuals from the case cohort were excluded from the study before analysis due to reasons outlined earlier in the materials and methods section.

Site of onset data from six individuals within the post-mortem cohort was recorded as ‘unknown’ which reflects informative missingness (e.g. nonresponse or not provided). Therefore, missingness was assumed to be not completely at random. A fully complete covariate dataset was available for the MND Register Cox proportional hazards regression and case-control logistic regression analysis.

## Results

### Age at symptom onset is decreased in highly active individuals with ALS

After exclusion, there were 139 ALS cases from the post-mortem cohort and 166 cases from the MND Register cohort available for full analysis. Both groups showed similar demographics (Table 1).

**Table 1:** Comparison of demographics in ALS cases from the post-mortem and MND Register Cohorts

Demographic	Post-mortem ALS cohort (*n* = 139)	MND Register ALS cohort (*n* = 166)	Test statistic (df)	*P*-value (test)	Effect size (test, 95% CI)
Sex ratio, male:female % (*n*)	60:40 (84:55)	60:40 (99:67)	χ^2^ (2) = 0.00	0.98 (χ^2^)	*ν* = 0.00 (Cramer’s *ν*, 0.00, 1.00)
Site of onset % (*n*)					
Bulbar	27 (37)	22 (36)	n/a	0.47 (Fisher’s exact)	*ν* = 0.00 (Cramer’s *ν*, 0.00, 1.00)
Spinal	69 (96)	75 (125)			
Unknown	4 (6)	3 (5)			
*C9orf72* mutation cases (*n*)	(13)	(3)	n/a	n/a	n/a

Post-mortem ALS cohort						
Demographic	Highly active (*n* = 16)	Active (*n* = 34)	Inactive (*n* = 89)	Test statistic (df)	*P*-value (test)	Effect size (test, 95% CI)
Sex ratio, male:female % (*n*)	88:12 (14:2)	62:38 (21:13)	55:45 (49:40)	n/a	0.04 (Fisher’s exact)	*ν* = 0.17 (Cramer’s *ν*, 0.00, 1.00)
Site of onset % (*n*)						
Bulbar	19 (3)	29 (10)	27 (24)	n/a	0.85 (Fisher’s exact)	*ν* = 0.00 (Cramer’s *ν*, 0.00, 1.00)
Spinal	81 (13)	65 (22)	68 (61)			
Unknown	0 (0)	6 (2)	5 (4)			
Mean age at onset^a,c^ (SD)	54.2 (7.5)	59.3 (11.5)	63.9 (11.5)	*F*(2, 136) = 6.10	0.003 (ANOVA)	$\eta^{2}$ = 0.08 (eta^2^, 0.02, 1.00)
Median age at onset^a,c^ (IQR)	55.0 (10.5)	62.0 (14.5)	65.0 (16)			
Mean disease duration^b,c^ (SD)	50.4 (18.6)	36.2 (19.0)	32.5 (20.8)			
Median disease duration^b,c^ (IQR)	45.0 (31.8)	32.0 (19.2)	28.0 (27.0)	*H*(2) = 12.30	0.002 (Kruskal–Wallis)	$\eta^{2}$ = 0.08 (eta^2^, 0.02, 0.17)

MND Register ALS Cohort						
Demographic	Highly active (*n* = 36)	Active (*n* = 107)	Inactive (*n* = 23)	Test statistic (df)	*P*-value (test)	Effect size (test, 95% CI)
Sex ratio, male:female % (*n*)	75:25 (27:9)	59:41 (63:44)	39:61 (9:14)	χ^2^ (2) = 7.57	0.02 (χ^2^)	*ν* = 0.18 (Cramer’s *ν*, 0.00, 1.00)
Site of onset % (*n*)						
Bulbar	19 (7)	18 (20)	39 (9)	n/a	0.18 (Fisher’s exact)	*ν* = 0.10 (Cramer’s *ν*, 0.00, 1.00)
Spinal	81 (29)	77 (82)	61 (14)			
Unknown	0 (0)	5 (5)	0 (0)			
Mean age at onset^a,c^ (SD)	56.7 (9.9)	61.1 (10.3)	64.5 (12.3)	n/a	n/a	n/a
Median age at onset^a,c^ (IQR)	58.0 (15)	63.0 (11)	63.0 (17.5)	*H*(2) = 7.39	0.02 (Kruskal–Wallis)	$\eta^{2}$ = 0.03 (eta^2^, −0.003, 0.12)

^a^Age at onset is defined as age at first recorded symptom (years).

^b^Disease duration is defined as symptom onset to death (months).

^c^Continuous parametric variables were compared by one-way ANOVA and continuous non-parametric variables were compared by a Kruskal–Wallis test.

Each cohort was split into ‘Highly Active’, ‘Active’ and ‘Inactive’ groups, revealing a significant shift towards male sex in the highly active groups. For the post-mortem cohort, a Fisher’s exact test revealed a significant association between sex ratio and physical activity group [*P* = 0.04, Cramer’s *ν* = 0.17 (small), 95% CI 0.00–1.00; Table 1], with a $\chi^{2}$ test of independence demonstrating the same significant relationship in the MND Register cohort [$\chi^{2}$ (2) = 7.57, *P* = 0.02, Cramer’s *ν* = 0.18 (small), 95% CI 0.00–1.00; Table 1]. However, upon conducting *post hoc* pairwise comparisons (Bonferroni correction) of the sex ratio in the post-mortem cohort, all results are non-significant (Highly Active; Inactive, *P* = 0.02, *P*.adj=0.07; Supplementary Table 1).

**Figure 1:**
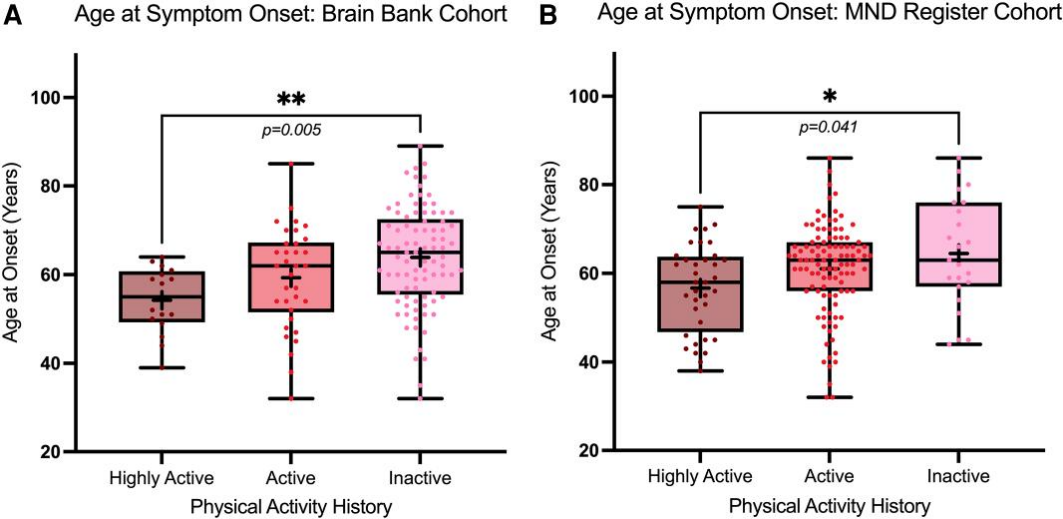
A decrease in age of symptom onset in highly active ALS patients. (**A**) Box plot of onset age in ALS cases grouped by physical activity history within the post-mortem cohort [Highly active (*n* = 16), Active (*n* = 34), Inactive (*n* = 89), *F*(2, 136) = 6.10, *P* = 0.003, $\eta^{2}$ = 0.08, 95% CI 0.02–1.00, one-way ANOVA; Tukey’s honestly significant difference for Inactive versus Highly active, M_diff = 9.65 *P* = 0.005, 95% CI 2.49–16.80]. (**B**) Box plot of onset age in ALS cases grouped by physical activity history within the Motor Neurone Disease (MND) Register cohort [Highly active (*n* = 36), Active (*n* = 107), Inactive (*n* = 23), *H*(2) = 7.39, *P* = 0.02, $\eta^{2}$ = 0.03, 95% CI 0.003–0.12, Kruskal–Wallace rank sum; Dunn–Bonferroni for Inactive versus Highly active, M_rank_diff = −2.47, *P*.adj = 0.04]. Box plots show median and interquartile ranges with individual data points plotted representing each person with ALS. Mean value is indicated by+.

Interestingly, both cohorts showed a strikingly similar significant difference in mean and median age at symptom onset between groups, with the highly active subgroup having the youngest age at disease onset [post-mortem: M = 54.2 years (SD 7.5), one-way ANOVA, *F*(2, 136) = 6.10, *P* = 0.003, $\eta^{2}$ = 0.08 (medium), 95% CI 0.02–1.00. MND Register: Mdn = 58 years (IQR 15), *H*(2) = 7.39, *P* = 0.02, $\eta^{2}$ = 0.03 (small), 95% CI −0.003–0.12; Table 1, Fig. 1]. Tukey’s honestly significant difference *post hoc* test for the post-mortem cohort revealed a significant difference between inactive and highly active groups (M_diff = 9.65 *P* = 0.005, 95% CI 2.49–16.80; Supplementary Table 2, Fig. 1). For the MND Register cohort, pairwise comparisons using Dunn’s *post hoc* test with Bonferroni correction also demonstrated a significant difference between inactive and highly active groups (M_rank_diff = −2.47, *P*.adj = 0.04; Supplementary Table 2, Fig. 1).

Median disease duration was not calculated for the MND Register cohort as some data points were censored, however, a significant difference in disease duration was seen between groups in the post-mortem cohort [*H*(2) = 12.30, *P* = 0.002, $\eta^{2}$ = 0.08 (medium), 95% CI 0.02–0.17; Table 1]. Full pairwise comparison results can be found in Supplementary Tables 1–3.

### Highly active individuals associated with younger ALS onset but not increased hazard of mortality

To assess the impact of strenuous physical activity on age at symptom onset and survival while controlling for other covariates such as sex, head injury and *C9orf72* expansion mutation, a Cox proportional hazards regression analysis was conducted.

**Figure 2:**
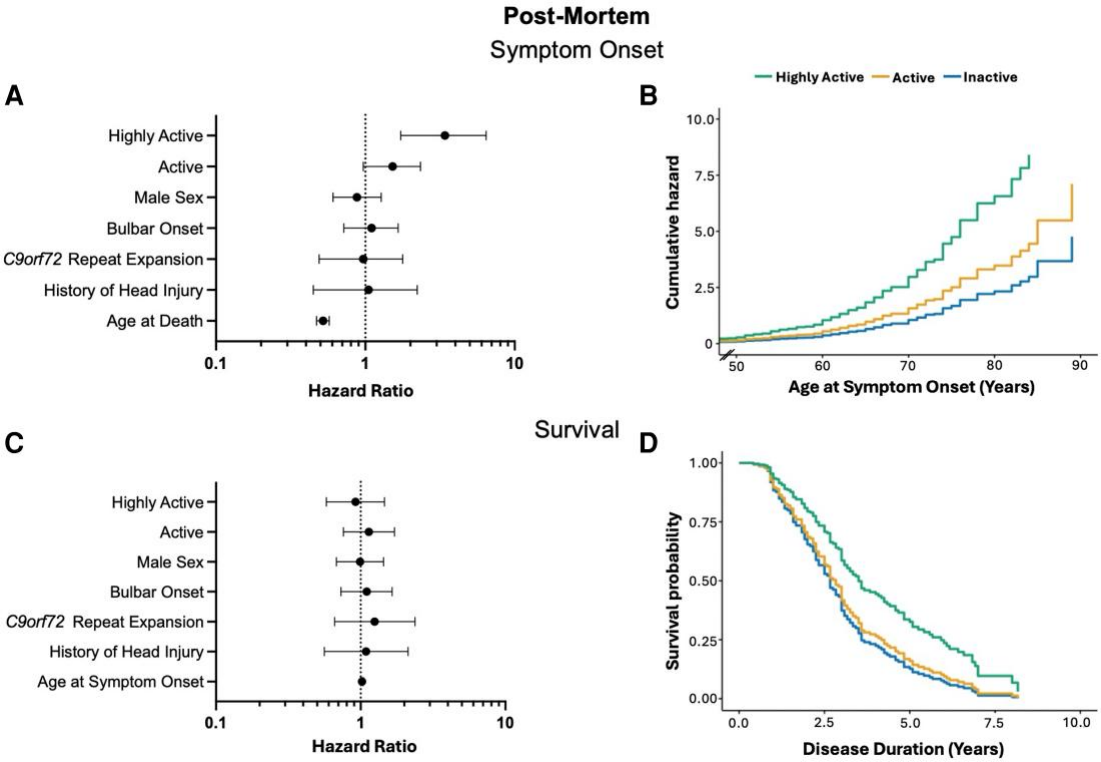
Cumulative hazard and survival analyses in the post-mortem cohort by physical activity history. (**A-B**) Highly active individuals (*n* = 16) had a significantly increased hazard of ALS symptom onset (HR 2.84, 95% CI 1.55–5.26, *P* = 0.0008; Cox proportional hazards regression). Active individuals (*n* = 34) showed a trend towards earlier onset that did not reach significance (HR 1.50, 95% CI 0.99–2.27, *P* = 0.062), compared to inactive individuals (*n* = 89). (**C-D**) No significant associations were observed between physical activity level and survival time: highly active (*n* = 16) (HR 0.92, 95% CI 0.58–1.46, *P* = 0.725) and active (*n* = 34) (HR 1.14, 95% CI 0.76–1.76, *P* = 0.535), in comparison to inactive (*n* = 89). Forest plots display hazard ratios with 95% confidence intervals.

In the post-mortem cohort, our multivariable model revealed that being a highly active individual was significantly associated with a higher risk of ALS symptom onset at a younger age [HR: 2.84, 95% CI 1.55–5.26, *P* = 0.0008; Table 2, Fig. 2A-B]. Active individuals also showed an increased HR for age at symptom onset; however, this was not significant (HR: 1.50, 95% CI 0.99–2.27, *P* = 0.054; Table 2, Fig. 2A-B). *C9orf72* repeat expansion mutation was significantly associated with onset risk (HR: 2.40, 95% CI 1.26–4.58, *P* = 0008; Table 2, Fig. 2A-B) but no other variables in the model demonstrated a significant relationship. When an alternative model was run including the LPA score rather than physical activity groups, the LPA score was significantly associated with age of symptom onset (HR: 1.03, 95% CI 1.01–1.06, *P* = 0.001) therefore, for every 1-unit increase in the LPA score, the risk of symptom onset increases by 3%. A multiple linear regression approach also yielded similar results [*F*(4, 134) = 5.165, *P* = 0.0007, adjusted R^2^ = 0.12)], with the LPA score being a significant predictor of onset age (β = −0.25, *P* = 0.037).

**Table 2:** Cox proportional hazards regression to assess the impact of strenuous physical activity and other covariates on age at symptom onset and survival in each cohort

Post-mortem ALS cohort: age at symptom onset			
Variable	*n*	HR (95% CI)^a^	*P*-value
Highly active^b^	16	2.84 (1.55–5.26)	0.0008
Active^b^	34	1.50 (0.99–2.27)	0.054
Male sex	84	1.22 (0.85–1.76)	0.275
*C9orf72* repeat expansion	13	2.40 (1.26–4.58)	0.008
History of Head Injury^c^	11	0.97 (0.82–1.14)	0.690

Post-mortem ALS cohort: survival			
Variable	*n*	HR (95% CI)^a^	*P*-value
Highly active^b,c^	16	0.92 (0.58–1.46)	0.725
Active^b,c^	34	1.14 (0.76–1.76)	0.535
Male sex	84	0.99 (0.68–1.44)	0.950
Bulbar onset	37	1.10 (0.73–1.65)	0.642
*C9orf72* repeat expansion	13	1.25 (0.66–2.37)	0.495
History of head injury	11	1.09 (0.56–2.13)	0.801
Age at symptom onset	139	1.02 (1.00–1.04)	0.022

MND Register ALS cohort: age at symptom onset			
Variable	*n*	HR (95% CI)^a^	*P*-value
Highly active^b^	36	2.34 (1.30–4.23)	0.005
Active^b^	107	1.52 (0.93–2.50)	0.095
Male sex	99	1.40 (1.00–1.97)	0.052
*C9orf72* repeat expansion	3	1.54 (0.48–4.93)	0.469
History of head injury	45	0.99 (0.70–1.42)	0.969

MND Register ALS cohort: survival			
Variable	*n*	HR (95% CI)^a^	*P*-value
Highly active^b^	22	1.19 (0.49–2.89)	0.710
Active^b^	74	1.65 (0.82–3.30)	0.157
Male sex	74	1.11 (0.67–1.82)	0.685
Bulbar onset	23	2.49 (1.36–4.57)	0.003
*C9orf72* repeat expansion	3	2.47 (0.57–10.66)	0.227
History of head injury	35	0.99 (0.59–1.67)	0.972
Age at symptom onset	111	1.07 (1.04–1.10)	6.95e−06

^a^HR, hazard ratio; CI, confidence interval.

^b^For physical activity groups, the Inactive group was set as the reference.

^c^Hazard ratios were calculated over time to ensure that the proportional hazard assumption was met.

A Cox proportional hazards regression was performed to evaluate the effect of multiple covariates on survival in the post-mortem ALS cohort. Neither the highly active (HR 0.92, 95% CI 0.58–1.46, *P* = 0.725) nor the active group (HR 1.14, 95% CI 0.76–1.76, *P* = 0.535) were significantly associated with survival when compared with inactive individuals (Table 2, Fig. 2C-D). Only age at symptom onset was significantly associated with hazard of mortality (HR: 1.02, 95% CI 1.00–1.04, *P* = 0.022; Table 2, Fig. 2C-D), in line with the established association between age at symptom onset and survival time in ALS.^34^ In the alternative LPA score model of survival, the LPA score was not significantly associated with survival in the post-mortem cohort (HR: 1.00, 95% CI 0.98–1.01, *P* = 0.717).

**Figure 3:**
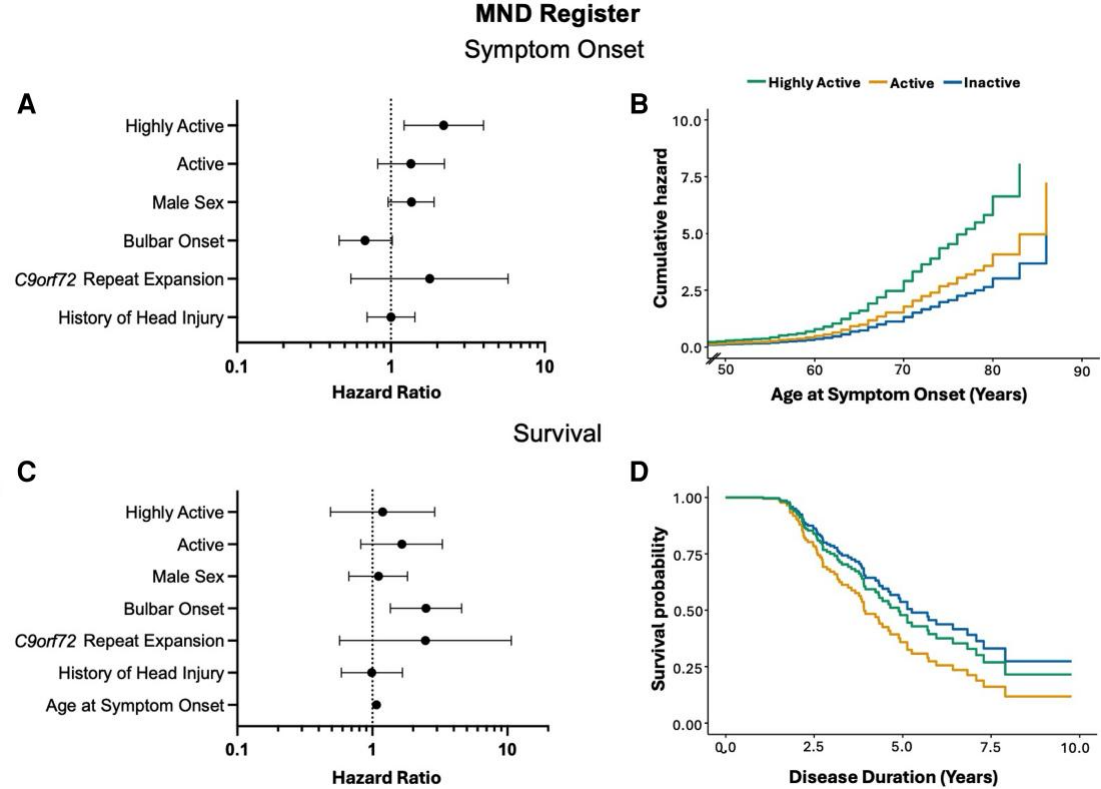
Cumulative hazard and survival analyses in the Motor Neurone Disease (MND) Register Cohort by physical activity history. (**A-B**) Highly active individuals (*n* = 36) had a significantly increased hazard of ALS symptom onset (HR 2.34, 95% CI 1.30–4.23, *P* = 0.005; Cox proportional hazards regression). Active individuals (*n* = 107) also showed an increased HR, but this was not significant (HR 1.52, 95% CI 0.93–2.50, *P* = 0.095), compared to inactive individuals (*n* = 23). (**C-D**) No significant associations were observed between physical activity level and survival time: highly active (*n* = 36) (HR 1.19, 95% CI 0.49–2.89, *P* = 0.710) and active individuals (*n* = 107) (HR 1.65, 95% CI 0.82–3.30, *P* = 0.157), in comparison to inactive (*n* = 23). Forest plots display HRs with 95% confidence intervals.

In the MND Register, highly active individuals had a significantly higher risk of ALS symptom onset at a younger age (HR 2.34, 95% CI 1.30–4.23, *P* = 0.005; Table 2, Fig. 3A–B). Active individuals also showed an increased HR, although this was not significant (HR 1.52, 95% CI 0.93–2.50, *P* = 0.095; Table 2, Fig. 3A–B). No other variables were significantly associated with onset risk in this cohort (Table 2). *C9orf72* repeat expansion was likewise not significantly associated with a higher risk of onset at a younger age (HR 1.54, 95% CI 0.48–4.93, *P* = 0.469; Table 2, Fig. 3A–B), although the small number of cases (three) within the cohort precludes firm conclusions.

When the Cox proportional hazards regression was run with the LPA score, the LPA score was significantly associated with age of symptom onset (HR: 1.02, 95% CI 1.00–1.03, *P* = 0.007) therefore, for every 1-unit increase in the LPA score, the risk of symptom onset increases by 2%. A multiple linear regression approach demonstrated an association between the LPA score and age at symptom onset in the MND Register cohort (*F*(5, 160) = 2.115, *P* = 0.06, adjusted *R*^2^ = 0.03), with the LPA score being a significant predictor of onset age (*β* = −0.13, *P* = 0.028).

Survival in the MND Register cohort was assessed using Cox proportional hazards regression. Neither highly active (HR 1.19, 95% CI 0.49–2.89, *P* = 0.710) nor active individuals (HR 1.65, 95% CI 0.82–3.30, *P* = 0.157) were significantly associated with hazard of mortality (Table 2, Fig. 3C-D). In contrast, bulbar onset was associated with a significantly higher hazard of mortality (HR 2.49, 95% CI 1.36–4.57, *P* = 0.003; Table 2; Fig. 3C-D), as was older age at symptom onset (HR 1.07, 95% CI 1.04–1.10, *P* = 6.95 *×*  *10^−6^*; Table 2; Fig. 3C-D).

In the model including the LPA score, the LPA score was not significantly associated with survival in the MND Register cohort (HR: 0.99, 95% CI 0.98–1.01, *P* = 0.244).

### Strenuous exercise is associated with a younger age of ALS onset but not increased risk of disease

There were 166 ALS cases and 196 controls from the MND Register MNDA epidemiology study cohort with full data available for analysis (Table 3).

**Table 3:** Comparison of case versus control cohort demographics from the MND Register

Demographic/behavioural measure	Case (*n* = 166)	Control (*n* = 196)	*P*-value^a^
Sex ratio, male:female % (*n*)	60:40 (99:67)	56:44 (110:99)	0.21
Median age at survey (IQR)	63.4 (12.7)	65.5 (11.8)	0.10
Median LPA score (IQR)	15.5 (19.5)	14.5 (18.2)	0.42
Highly active individuals % (*n*)	21.7 (36)	17.3 (34)	0.36
Active individuals % (*n*)	64.4 (107)	64.3 (126)	1.00
Inactive individuals % (*n*)	13.9 (23)	18.4 (36)	0.31
Median age exercise initiation (*n*)	11.5 (9)	12 (9)	0.87
Median total years exercising (*n*)	36.0 (37)	38.5 (41.5)	0.64
History of head injury % (*n*)	27.1 (45)	17.3 (34)	0.03

^a^Mann–Whitney U tests were used for continuous variables and $\chi^{2}$ for categorical variables as they were non-normally distributed.

The case group showed a marginally higher median LPA score (15.5, IQR 19.5) compared with controls (14.5, IQR 18.2) and a higher proportion of highly active individuals (21.7% versus 17.3%), but neither difference was significant (Mann–Whitney U and $\chi^{2}$ tests). There were also no significant differences in median age of exercise initiation (cases: 11.5 years; controls: 12 years) or total years of exercise (cases: 36.0 years; controls: 38.5 years). By contrast, a history of head injury was significantly more common in people with ALS (27.1%) than in controls (17.3%, *P* = 0.03).

Logistic regression adjusted for age at survey, sex and head injury confirmed no significant association between physical activity and ALS risk (Table 4). Highly active individuals had an OR of 1.43 (95% CI 0.69–2.99, *P* = 0.333), while active individuals had an OR of 1.30 (95% CI 0.72–2.37, *P* = 0.386). In contrast, a history of head injury was significantly associated with ALS risk (OR 1.72, 95% CI 1.03–2.88, *P* = 0.038).

**Table 4:** Logistic regression model for physical exercise and risk of ALS

Variable	*n*	OR (95% CI)^a^	*P*-value
Highly active^b^	70	1.43 (0.69–2.99)	0.333
Active^c^	233	1.30 (0.72–2.37)	0.386
Male sex	209	1.01 (0.65–1.57)	0.964
Age at survey	362	0.99 (0.97–1.01)	0.244
History of head injury	79	1.72 (1.03–2.88)	0.038

^a^OR, odds ratio; CI, confidence interval.

^b,c^For physical activity groups, the Inactive group was set as the reference.

## Discussion

Overall, our analysis suggests a younger age of symptom onset of up to ∼10 years, in highly active individuals with ALS, validated in two similar but distinct patient cohorts in the UK. The strikingly similar results observed in both patient groups strengthens the evidence for a relationship between strenuous physical activity and ALS onset and is consistent with previous findings which have suggested a decreased age of disease onset in other ALS athlete populations.^22,24,25^ We propose that there is a dose dependent effect of exercise, where minimal to moderate activity levels have little to no impact on reducing age of onset, until a threshold level is reached, when strenuous exercise may trigger disease at an earlier age. This is supported by our findings where active individuals display an increased HR without reaching statistical significance (post-mortem: HR 1.50, 95% CI 0.99–2.27, *P* = 0.054; MND Register: HR 1.52, 95% CI 0.93–2.50, *P* = 0095).

Physical activity was not significantly associated with ALS disease risk (Highly active individuals: OR 1.43, 95% CI 0.69–2.99, *P* = 0.333, active individuals OR 1.30, 95% CI 0.72–2.37, *P* = 0.386), although, both active groups showed an increased odds ratio. Interestingly, our results reveal a significant association between a history of head injury and an increased risk of ALS (OR 1.72, 95% CI 1.03–2.88, *P* = 0.038), as has been suggested in previous research.^13-15^ These findings further highlight the complex relationship between physical activity, head injury (including repetitive mild head injury from contact sports) and disease risk. Indeed, we did not see any relationship between a history of head injury and a younger age of symptom onset in either ALS patient cohort (post-mortem: HR 0.97, 95% CI 0.82–1.14, *P* = 0.690; MND Register: HR 0.99, 95% CI 0.70–1.42, *P* = 0.969). Therefore, we propose that strenuous exercise alone may be associated with early-onset ALS, possibly in predisposed individuals, whereas head injury may be related to risk of ALS development or associated through reverse causation. We highlight the importance of acknowledging ALS incidence and onset as a multistage process,^10^ whereby environmental factors such as physical exercise and head injury may each contribute steps.

A recent study which analysed UK electronic health records from over 85 000 individuals reported an increased risk of ALS in those with documented traumatic brain injury (HR 2.61, 95% CI 1.88–3.63, *P* < 0.001).^16^ Interestingly, this association was time-dependent, being strongest within the first 2 years after injury and then attenuating thereafter.^16^ Similarly to our study, these findings support the association between ALS and head injury at a population level. However, the association of head injury and increased risk of ALS must be interpreted with caution as it may in fact indicate reverse causality.^16^ Rather than traumatic brain injury triggering the neurodegenerative process of ALS, traumatic brain injury may instead arise from early subclinical symptoms of ALS where individuals are more at risk of falls. Similarly, another group using the Oxford Record Linkage Study found an adjusted rate ratio of 1.5 (95% confidence interval 1.1–2.1) for ALS after head injury, compared with a control group, however, this increased risk was only present within the first year after injury.^35^ Several studies have reported the delay between demonstration of first symptoms and ALS diagnosis, and research suggests that the neurodegenerative process starts decades earlier than symptoms manifest.^36^

Our study population includes highly active individuals who are, to the best of our knowledge, not professional athletes, although many participated in high-level sporting activities. This makes our findings replicable to the general population rather than specific to professional sportspeople with unique lifestyle habits. Additionally, we explore the impact of overall energy expenditure in the form of MET scores across many different types of exercise (both anaerobic and aerobic), producing a more comprehensive review of activity levels rather than focusing solely on one sport. Interestingly, we see a higher proportion of men in the highly active group in both cohorts (post-mortem: 88%, MND Register: 75%). Although male sex alone was not associated with a younger age of symptom onset, our findings raise questions regarding whether males may be more commonly affected by exercise-related-ALS, as recently proposed.^25^

It has been suggested that people with ALS may be more active overall than controls, raising questions as to whether it is exercise itself that is responsible for an association with ALS, or rather a biological profile which is advantageous for exercise but also has a related increased risk of disease onset. Although we cannot rule this out as a possibility, our results from the case-control analysis suggest that individuals with ALS are not significantly more active than age- and sex-matched controls (median LPA score in case group: 15.5, IQR 19.5, compared with controls, 14.5, IQR 18.2; *P* = 0.42, Mann–Whitney U), and thus that it is the exercise itself that is associated with symptom onset.

We did not have a large enough number of *C9orf72* expansion mutation cases in either cohort to perform an in-depth investigation into the relationship between genetics and environmental risk factors. Future research should focus on any association between common ALS genetic mutations, exercise, age at onset and the impact of head injury.

### Limitations

Physical activity data were retrospectively collected from clinical records in the post-mortem cohort and by self-reported questionnaire in the MND Register cohort. Both approaches are vulnerable to recall bias and may lead to under- or overestimation of lifetime activity. We also acknowledge that the resulting LPA score for each donor is a measure of total energy expenditure based on activity type and, due to incomplete exercise frequency data, may be limited in capturing the full scope of the exercise undertaken. Despite this, our study demonstrates an association between a younger age of onset and the most strenuous exercisers, but questions remain as to whether engaging in more strenuous activity for a shorter duration or less strenuous activity for a longer time period changes this association. Previous studies have highlighted the importance of anaerobic versus aerobic exercise^18,26,37^ and their individual links to ALS. Therefore, future research should incorporate activity type, anaerobic versus aerobic exercise, frequency and duration data.

The association of higher exercise levels with an earlier age of onset might be influenced by better recall of more intense physical activity by younger individuals. Additionally, those with an older age of symptom onset will have had more cumulative time to exercise throughout their life. Thus, these factors must be considered as a confound when interpreting the case-control analysis.

The study cohorts were derived from UK populations, which may limit the generalizability of the findings to other populations. In particular, the relatively small sample sizes in subgroup analyses reduce power to detect modest effects, especially for less common variables such as whether someone carried *C9orf72* expansion or had a history of head injury. The small number of mutation carriers precluded detailed analyses of gene–environment interactions, which remain an important area for future research.

Survival analyses in the MND Register were affected by censoring due to incomplete follow-up, and disease duration could not be reliably calculated in this cohort. Although Cox regression accounts for censored data, the absence of complete follow-up may have reduced sensitivity to detect survival differences. While we adjusted for known covariates such as age, sex, site of onset and head injury, unmeasured confounders, including lifestyle, occupational exposures or comorbidities, may have influenced the results. Future studies with larger, more diverse cohorts and prospective designs are needed to validate these findings and explore the biological mechanisms linking physical activity, head injury and ALS onset.

We were unable to compare data from healthy controls in the post-mortem cohort as they were not routinely asked about their physical activity levels, in comparison to the ALS patients who were more likely to be questioned on their exercise abilities prior to symptom onset by clinicians.

Despite these limitations, the concordance of findings across both groups strongly supports the hypothesis that physical activity does have a role to play in ALS onset.

## Conclusion

Our study suggests a younger age of symptom onset in highly active individuals with ALS, adding weight to the evidence that strenuous exercise may contribute to an earlier disease onset in susceptible people. Future research should investigate the impact of genetic risk factors in the relationship between ALS and exercise as well as considering potential pathophysiological mechanisms which may be involved in symptom onset. Pathways known to be impacted in ALS, such as energy metabolism, oxidative stress and glutamate excitotoxicity, should be explored in the context of intensive exercise to identify dysregulated responses which may accelerate disease onset. Understanding pathways important in driving disease onset, both within the response to exercise but also the initiation of ALS as a whole, would enable the identification of potential new therapeutic targets.

Importantly, our results reveal that a history of head injury was significantly associated with ALS risk but not a younger age of symptom onset. We suggest that strenuous exercise may accelerate disease onset in predisposed individuals, while head injury may contribute independently to disease susceptibility, or be associated through reverse causality. Identifying both environmental risk factors and disease modifiers for ALS is essential to advancing preventative strategies and developing personalized therapies for individuals most at risk.

## Supplementary Material

fcag272_Supplementary_Data

## Data Availability

Access to the MND register can be requested here https://mndregister.ac.uk and access to the MND Association MND Collections here https://www.mndassociation.org/research/our-research/uk-mnd-collections-samples. Data derived from the Brain Bank can be accessed by request to the authors. All R code generated for this manuscript has been uploaded to an online repository and can be accessed using the following link: https://doi.org/10.5281/zenodo.18508813.
