# Supplementary material for: Strenuous physical activity is associated with a younger age of amyotrophic lateral sclerosis onset in two independent cohorts
Source: Brain Commun. 2026 Jul 13;8(4):fcag272. doi: 10.1093/braincomms/fcag272 (PMC13392466; doi:10.1093/braincomms/fcag272)
Supplement: fcag272_Supplementary_Data [file fcag272_supplementary_data.pdf]

Supplementary Material

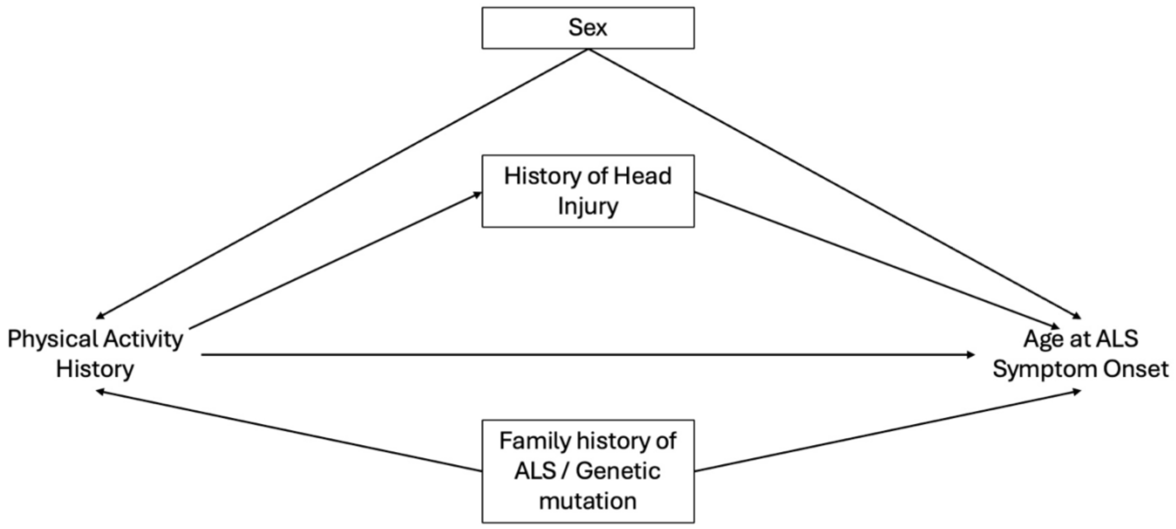

Supplementary Figure 1. Directed Acyclic Graph demonstrating the associations between physical activity history and age at ALS symptom onset in the Post-Mortem and Motor Neurone Disease (MND) Register Cohorts.

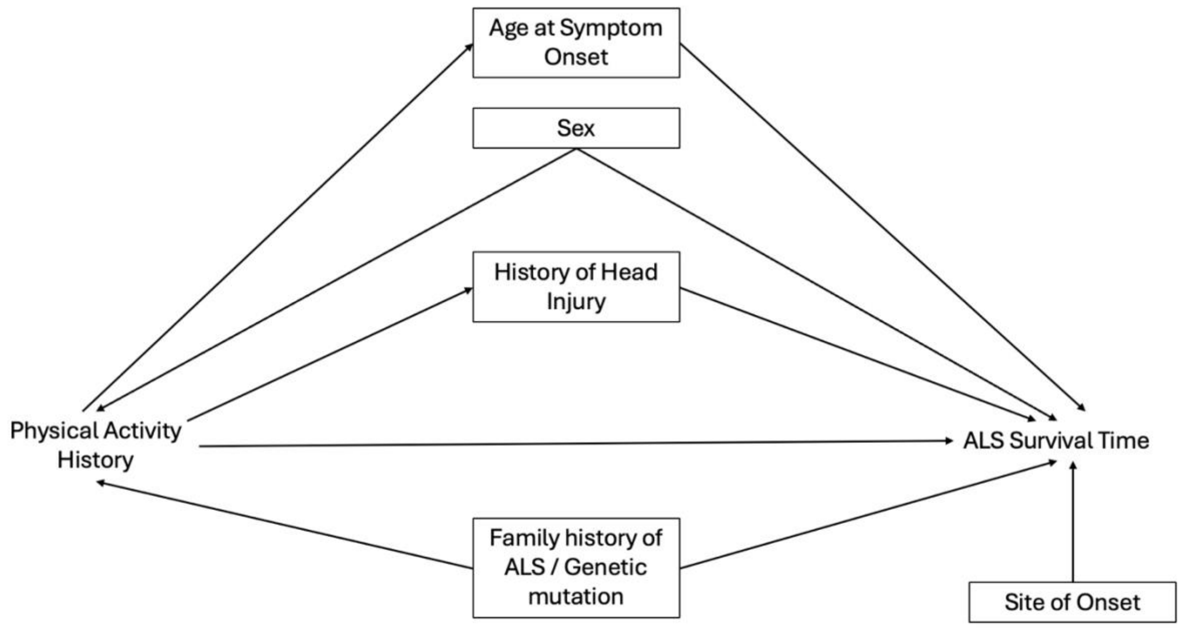

Supplementary Figure 2. Directed Acyclic Graph demonstrating the associations between physical activity history and ALS survival time in the Post-Mortem and Motor Neurone Disease (MND) Register Cohorts.

Supplementary Table 1. Pairwise Comparisons of ALS sex ratio by physical activity history from the Post-Mortem Cohort.

Post-Mortem ALS Cohort (Fisher's exact, Bonferroni)

Group 1	Group 2	P-value	P.adj	P.signif
Inactive (n=89)	Active (n=34)	0.546	1.000	ns
Inactive (n=89)	Highly Active (n=16)	0.024	0.072	ns
Active (n=34)	Highly Active (n=16)	0.090	0.297	ns

MND Register ALS Cohort (Chi-squared, FDR[a])

Group 1	Group 2	P-value	P.adj	P.signif
Inactive (n=89)	Active (n=34)	0.084	0.084	ns
**Inactive (n=89)**	**Highly Active (n=16)**	**0.006**	**0.018**	*
Active (n=34)	Highly Active (n=16)	0.080	0.080	ns

[a]FDR = False Discovery Rate

Supplementary Table 2. Pairwise Comparisons of ALS onset age by physical activity history from the Post-Mortem and MND Register Cohorts.

Post-Mortem ALS Cohort (One-way ANOVA[a], Tukey's HSD[b])

Group 1	Group 2	Estimate	Conf. low	Conf. High	P.adj	P.signif
Inactive (n=89)	Active (n=34)	4.58	-0.740	9.89	0.107	ns
**Inactive (n=89)**	**Highly Active (n=16)**	**9.65**	**2.49**	**16.80**	**0.005**	**
Active (n=34)	Highly Active (n=16)	5.07	-2.92	13.10	0.292	ns

MND Register ALS Cohort (Kruskal-Wallis, Dunn-Bonferroni)

Group 1	Group 2	Statistic[c]	P-value	P.adj	P.signif
Inactive (n=23)	Active (n=107)	-0.94	0.347	1.00	ns
**Inactive (n=23)**	**Highly Active (n=36)**	**-2.47**	**0.010**	**0.04**	*
Active (n=107)	Highly Active (n=36)	-2.29	0.020	0.07	ns

[a]ANOVA = Analysis of Variance
[b]HDR = Honestly Significant Difference
[c]Dunn-Bonferroni statistics represent standardized differences in mean ranks; negative values indicate lower onset age in group 2.

Supplementary Table 3. Pairwise Comparisons of ALS disease duration by physical activity history from the Post-Mortem Cohort.

Post-Mortem ALS Cohort (Kruskal-Wallis, Dunn-Bonferroni)

Group 1	Group 2	Statistic[a]	P-value	P.adj	P.signif
Inactive (n=89)	Active (n=34)	1.24	0.215	0.646	ns
**Inactive (n=89)**	**Highly Active (n=16)**	**3.47**	**0.0005**	**0.0002**	**
Active (n=34)	Highly Active (n=16)	2.28	0.023	0.068	ns

[a]Dunn-Bonferroni statistics represent standardized differences in mean ranks; positive values indicate longer disease duration in group 2.
